# Location of Pathogenic Bacteria during Persistent Infections: Insights from an Analysis Using Game Theory

**DOI:** 10.1371/journal.pone.0005383

**Published:** 2009-04-29

**Authors:** Sandeepa M. Eswarappa

**Affiliations:** Centre for Infectious Disease Research and Biosafety Laboratories, Department of Microbiology and Cell Biology, Indian Institute of Science, Bangalore, India; University of Hyderabad, India

## Abstract

Bacterial persistent infections are responsible for a significant amount of the human morbidity and mortality. Unlike acute bacterial infections, it is very difficult to treat persistent bacterial infections (e.g. tuberculosis). Knowledge about the location of pathogenic bacteria during persistent infection will help to treat such conditions by designing novel drugs which can reach such locations. In this study, events of bacterial persistent infections were analyzed using game theory. A game was defined where the pathogen and the host are the two players with a conflict of interest. Criteria for the establishment of Nash equilibrium were calculated for this game. This theoretical model, which is very simple and heuristic, predicts that during persistent infections pathogenic bacteria stay in both intracellular and extracellular compartments of the host. The result of this study implies that a bacterium should be able to survive in both intracellular and extracellular compartments of the host in order to cause persistent infections. This explains why persistent infections are more often caused by intracellular pathogens like *Mycobacterium* and *Salmonella*. Moreover, this prediction is in consistence with the results of previous experimental studies.

## Introduction

When a pathogenic bacterium invades a host, activation of innate and adaptive immune systems of the host ensues, which results in disease symptoms. The resulting host-pathogen interaction can lead to either the death of the host or the clearance of the pathogen depending on the nature of the pathogen. However, some pathogens continue to thrive inside mammalian hosts despite the robust antimicrobial activities of the host resulting in persistent infection. According to Blaser *et al*, “persistence represents the evolved selection for balancing host and microbial interests, resulting in an equilibrium that, by definition, is long-term but not necessarily forever stable” [1].

Bacterial persistent infections are major causes of human morbidity and mortality. Unfortunately, our knowledge about the basis of bacterial persistent infections is not adequate to effectively prevent and treat such infections. The best example for the bacterium that can cause persistent infection is *Helicobacter pylori*. More than half of the world population is having persistent infection with *H. pylori* in their gastric mucosa which causes peptic ulcer disease and is also an early risk factor for gastric cancer [2]. *Mycobacterium tuberculosis* can cause persistent infection in human lungs. This bacterium has infected one-third of the world population and many of these infected individuals will not show signs of tuberculosis in their life time [3]. *Salmonella enterica* serovar Typhi, *Brucella* spp., *Borrelia burgdorferi*, *Bartonella henselae*, *Neisseria gonorrhoea*, *Neisseria meningitidis*, *Streptococcus pneumoniae*, *Streptococcus pyogenes*, *Haemophilus influenzae* type B are some examples of bacterial pathogens which cause persistent infections in humans [4].

The compartment of the host in which pathogenic bacteria stay for considerably long time during persistent infection should ensure safety as well as easy transmissibility to the bacteria. Knowledge about such a location of pathogenic bacteria during persistent infections will help to find appropriate drugs which can reach those locations and also to design suitable vaccines. Thus, it is crucial for the disease management to understand the location of pathogenic bacteria during persistent infection. Many elegant theoretical studies have improved our understanding of persistent infections [5]–[9]. Although the location of some pathogenic bacteria (e.g. *H. pylori* and *M. tuberculosis*) during persistent infection is known [4], there is no generic theoretical model which predicts the same.

## Results

Interaction of a bacterial pathogen with a host is a clear case of conflict of interest. Bacteria aim to increase their payoff by multiplying as much as possible and then by getting transmitted out of the host as efficiently as possible to reach a new host. In contrast, the host tries to minimize its loss by eliminating the bacteria as early as possible with minimal investment and damage to itself. Broadly, there are two locations in which bacteria can thrive inside the host - extracellular compartment and intracellular compartment. Accordingly, to counteract the invading bacteria, the host can execute its extracellular defense mechanisms (e.g. antimicrobial peptides, complement system and antibodies) and/or intracellular defense mechanisms (e.g. reactive oxygen and nitrogen intermediates and lysosomes). During persistent infection, these host-pathogen interactions reach Nash equilibrium [1]. In a game, a set of strategies is a Nash equilibrium if no player can do better by unilaterally altering his strategies. This concept was first proposed by John Forbes Nash after whose name the equilibrium is named [10]. The above mentioned events of persistent infection can be analyzed using game theory to get more insights into the location of pathogenic bacteria inside the host; to achieve the same, the following game was defined.

### Definition of the game

#### 1. Players

The host (H) and the pathogen (bacteria in this study) (P)

#### 2. Strategies

(i) Strategies of the pathogenic bacteria:

- to thrive in extracellular compartment of the host (P_extra_)
- to thrive in intracellular compartment of the host (P_intra_)

*X* = (*x_1_*, *x_2_*) is the mixed strategy of the bacteria which implies that bacteria play P_extra_ with a probability of *x_1_* and P_intra_ with a probability of *x_2_* such that *x_1_*+*x_2_* = 1.

X^*^ = (x_1_^*^, x_2_^*^) is the mixed strategy of the bacteria during Nash equilibrium which implies that during Nash equilibrium, bacteria play P_extra_ with a probability of x_1_^*^ and P_intra_ with a probability of x_2_^*^ such that x_1_^*^+x_2_^*^ = 1.

(ii) Strategies of the host:

- to counteract bacteria using extracellular defense mechanisms (H_extra_)
- to counteract bacteria using intracellular defense mechanisms (H_intra_)

*Y* = (*y_1_*, *y_2_*) is the mixed strategy of the host which implies that the host plays H_extra_ with a probability of *y_1_* and H_intra_ with a probability of *y_2_* such that *y_1_*+*y_2_* = 1.

Y^*^ = (y_1_^*^, y_2_^*^) is the mixed strategy of the host during Nash equilibrium which implies that during Nash equilibrium, the host plays H_extra_ with a probability of y_1_^*^ and H_intra_ with a probability of y_2_^*^ such that y_1_^*^+y_2_^*^ = *1*.

#### 3. Utility or Payoff

**Figure 1:**
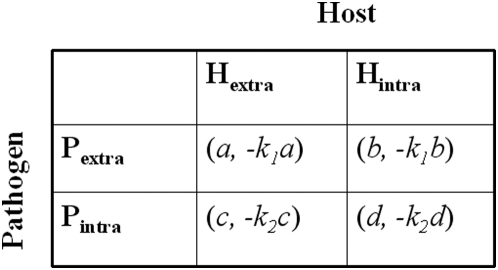
Payoff matrix of host-pathogen game. The game consists of two players- the host and the bacterial pathogen. P_extra_ and P_intra_ are the strategies of the bacterial pathogen to thrive inside the extracellular and intracellular compartments of the host, respectively. H_extra_ and H_intra_ are the strategies of the host to counteract the bacterial pathogen using extracellular and intracellular defense mechanisms, respectively. *a*, *b*, *c* and *d* are the payoffs of the bacterial pathogen during four possible pure strategy situations and *−k_1_a*, *−k_1_b*, *−k_2_c* and *−k_2_d* are the corresponding payoffs of the host. *k_1_* and *k_2_* are proportionality constants.

Payoff of each player is defined in the payoff matrix in Fig. 1.

Payoff of the pathogen (*U_P_*) is given by the following equation:
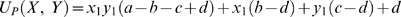
(1)


Payoff of the host (*U_H_*) is given by the following equation:
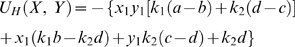
(2)Where, *a*, *b*, *c* and *d* are payoffs of bacteria in different situations (Fig. 1); *k_1_* and *k_2_* are proportionality constants; *X*, *Y*, *x_1_*, *x_2_*, *y_1_* and *y_2_* have already been defined above.

This game is based on few reasonable assumptions. I have assumed that all extracellular compartments (to which a particular bacteria is exposed) of a host are similar with respect to their anti bacterial activity and all intracellular compartments (to which a particular bacteria is exposed) are also similar with respect to their antibacterial activity. I have also assumed that the damage caused to the host is directly proportional to the gain of the bacteria (*U_H_* = *−kU_P_*, where *k* is a proportionality constant).

### Analysis of Nash Equilibria

Using *H. pylori*, *M. tuberculosis* and *S. typhi* as model pathogens, Blaser *et al.* have proposed that bacterial persistent infections represent a co-evolved series of nested equilibria, conforming to Nash equilibria, operating simultaneously in multiple levels (cellular, populational and evolutionary levels) [1]. Therefore, during persistent infection, according to the definition of Nash equilibrium,
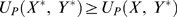
(3)and
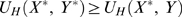
(4)Using these relations (Eq. 3 and Eq. 4) it is possible to compute the possible Nash equilibria of this game. Calculations revealed that there are three Nash equilibria in this game (see Supporting Information S1 for the details of calculation). They are as follows (Table 1):

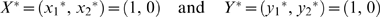

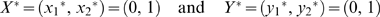

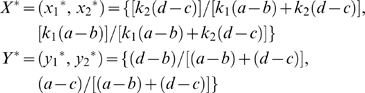

**Table 1:** Possible Nash equilibria of Host-Pathogen game.

	Nash equilibrium strategies	Condition	Remarks
**1**	X^*^ = (x_1_^*^, x_2_^*^) = (1, 0) and Y^*^ = (y_1_^*^, y_2_^*^) = (1, 0)	*a*>*c* & *a*<*b*	Biologically not plausible
**2**	X^*^ = (x_1_^*^, x_2_^*^) = (0, 1) and Y^*^ = (y_1_^*^, y_2_^*^) = (0, 1)	d>b & c>d	Biologically not plausible
**3**	X^*^ = (x_1_^*^, x_2_^*^) = {[*k_2_*(*d*−*c*)]/[*k_1_*(*a*−*b*)+*k_2_*(*d*−*c*)], [*k_1_*(*a*−*b*)]/[*k_1_*(*a*−*b*)+*k_2_*(*d*−*c*)]}	-	Biologically plausible
	and		
	Y^*^ = (y_1_^*^, y_2_^*^) = {(*d*−*b*)/[(*a*−*b*)+(*d*−*c*)], (*a*−*c*)/[(*a*−*b*)+(*d*−*c*)]}		

First Nash equilibrium, where x_1_^*^ = 1 and y_1_^*^ = 1, is possible when
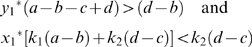
[See (Eq. A.2) and (Eq. A.7) in Supporting Information S1]

Substituting y_1_^*^ = 1 and x_1_^*^ = 1 in these inequalities, we get *a*>*c* and *a*<*b*.

‘*a*’ is the payoff of bacteria when bacteria thrives only in extracellular compartment and the host attacks bacteria using only extracellular defense mechanisms. ‘*b*’ is the payoff of bacteria when bacteria thrives only in extracellular environment, but the host attacks bacteria using only intracellular defense mechanisms. Therefore, *b*>*a* is biologically possible. ‘*c*’ is the payoff of bacteria when bacteria thrives only in intracellular compartment but the host attacks bacteria using only extracellular defense mechanisms. Therefore, *a*>*c* is not a biologically possible situation. This implies that the first Nash equilibrium, X^*^ = (x_1_^*^, x_2_^*^) = (1, 0) & Y^*^ = (y_1_^*^, y_2_^*^) = (1, 0), is biologically not plausible.

Second Nash equilibrium, where x_1_^*^ = 0 and y_1_^*^ = 0, is possible when
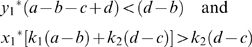
[See (Eq. A.3) and (Eq. A.6) in Supporting Information S1]

Substituting y_1_^*^ = 0 and x_1_^*^ = 0 in these inequalities, we get d>*b* and *c*>*d*.

‘*c*’ is the payoff of bacteria when bacteria thrives only in intracellular compartment and the host attacks bacteria using only extracellular defense mechanisms. ‘*d*’ is the payoff of bacteria when bacteria thrives only in intracellular environment and the host attacks bacteria using only intracellular defense mechanisms. Therefore, *c*>*d* is biologically possible. ‘*b*’ is the payoff of bacteria when bacteria thrives only in extracellular compartment but the host attacks bacteria using only intracellular defense mechanisms. Therefore, *d*>*b* is not a biologically possible situation. This implies that the second Nash equilibrium, X^*^ = (x_1_^*^, x_2_^*^) = (0, 1) and Y^*^ = (y_1_^*^, y_2_^*^) = (0, 1), is also biologically not plausible.

The third Nash equilibrium is unconditional [see (Eq. A.4) and (Eq. A.8)].
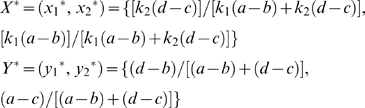
This condition defines the Nash equilibrium of this game.

(Results of this section are summarized in Table 1.)

### Conditions for persistent infection

From the analysis of Nash equilibria (see above), we can infer the following conditions for bacterial persistent infection:

1. 0<x_1_^*^<1
2. 0<x_2_^*^<1
3. 0<y_1_^*^<1
4. 0<y_2_^*^<1

To put in words, during persistent infection, bacteria stays in both extracellular and intracellular compartments of the host and the host counteracts the bacterial pathogen using both extracellular and intracellular defense mechanisms.

## Discussion

The location of pathogenic bacteria inside the host body during persistent infection is crucial for its success as a pathogen. Intracellular compartment is relatively safer if bacteria can avoid phagolysosomal fusion as it protects bacteria from immune recognition and serum mediated extracellular killing by the host system. In fact, many bacterial pathogens which cause persistent infections (*Mycobacterium*, *H. pylori*, *Salmonella*, *Brucella*, etc.) are known to inhibit phagolysosomal fusion [11]–[14]. In contrast, extracellular compartment will render bacteria an easy access to the external environment for dissemination (e.g. gastrointestinal tract for *Salmonella* and *H. pylori* and respiratory tract for *Mycobacterium*) which is also vital for a pathogen. An important disadvantage of intracellular location is that the bacteria can stay there only as long as the host cell is alive. Thus, pathogenic bacteria face a trade-off between ‘safety’ of intracellular compartment and relative ease of ‘dissemination’ via extracellular compartment. Predictions of the present theoretical model clearly indicate the presence of such a trade-off which ensues the localization of bacteria in both extracellular and intracellular compartments of the host during persistent infections.

The prediction of this model implies that a bacterium should be able to survive both in intracellular and extracellular compartments of the host in order to cause persistent infections. This explains, to some extent, why persistent infections are more often caused by intracellular pathogens like *Mycobacterium*, *Salmonella*, *Brucella* spp., *Neisseria gonorrhoea*, *Neisseria meningitides* etc [4]. Although *H*. *pylori* and *Streptococcus* Spp. which cause persistent infections have long been considered as extracellular pathogens, they also exploit intracellular compartment during persistence [4], [15], [16]. As intracellular pathogens have to cross extracellular compartments before reaching intracellular compartment, they must have evolved strategies to withstand the antimicrobial activities of both the compartments and therefore, they are more likely to cause persistent infections.

The analysis I have provided can not be extrapolated to any two locations in the host body. The results are applicable only for those locations (i) which are distinct from each other in their defense mechanisms, (ii) to which a pathogen is exposed, and (iii) where (y_1_+y_2_)≈1 is satisfied. Distinctness in the defense mechanisms ensures two (or more) different strategies for the host and exposure to such locations ensures two (or more) different strategies for the pathogen. Dividing a host into extracellular and intracellular compartments satisfy all these criteria. Apparently, it is hard to find any two compartments in the body which satisfy all these criteria. Therefore, the present analysis is specific to extracellular and intracellular environments.

Experimental studies on few bacteria which cause persistent infections clearly demonstrate that they localize in both extracellular and intracellular compartments of the host [4]. *H. pylori* is shown to be present in both extracellular and intracellular compartments of gastric mucosal layer [15]. The *H. pylori* population in the intracellular compartment acts as a store which repopulates the predominant extracellular population [17]. *Salmonella* persistence model in mice has demonstrated that although majority of the bacteria are intracellular, there will be some bacteria in the extracellular compartment also [18]. *Mycobacterium* is also known to stay in both extracellular and intracellular compartments in the granuloma [3]. Thus, prediction of the present theoretical model is in consistence with the previous experimental observations and it can be applied to any other bacterial pathogen which causes persistent infections. This theoretical model can also be extended to obligate intracellular pathogens like *Chlamydia* spp. by appropriate modifications to the structure of the game. However, this model can not be used to analyze interactions of the host with commensals. In commensalism, the commensal gains but the host neither gains nor loses. Therefore, there is no conflict of interest in commensalism and thus, game theory may not be an appropriate tool to explain the events in pure commensalism.

Unlike acute infections, persistent infections are difficult to treat owing to their complex biology which involves host-pathogen interactions at various levels ranging from molecular to population levels and maintenance of such interactions for prolonged time adds further complexity [19]. This difficulty is exemplified by the prolonged time (6–9 months) required to treat tuberculosis patients which is challenged by non-compliance and multidrug resistant bacteria [3]. Persistent infection of *Salmonella* (chronic carriers of *S.* Typhi) also requires a prolonged antibiotic treatment and some times surgical intervention to remove the gall bladder [20]. As the present theoretical model predicts, bacteria are present in both extracellular and intracellular environments during persistent infections and it might be sufficient to target population present in any one of these compartments to break the equilibrium and thus, the persistence. This therapeutic implication of the present theoretical model is very important considering the fact that it is difficult to target and eliminate intracellular pathogenic bacteria [21].

The present theoretical model is highly simplified to make the predictions more general and to allow heuristic understanding. This model can be extended by appropriate modifications for explicit representation of any specific host-pathogen interaction.

## Supporting Information

Supporting Information S1 Computation of the Nash equilibrium strategies of the host and the pathogen
(0.05 MB DOC)
