# Supplementary material for: Location of Pathogenic Bacteria during Persistent Infections: Insights from an Analysis Using Game Theory
Source: PLoS One. 2009 Apr 29;4(4):e5383. doi: 10.1371/journal.pone.0005383 (PMC2671611; doi:10.1371/journal.pone.0005383)
Supplement: Supporting Information S1 — Computation of the Nash equilibrium strategies of the host and the pathogen (0.05 MB DOC) [file pone.0005383.s001.doc]

**Supporting Information S1**

**Computation of the Nash equilibrium strategies of the host and the pathogen**

I. From (Eq.1) and (Eq.3) (see main text, Results section),

{*x_1_^*^y_1_^*^*(*a-b-c+d*)+*x_1_^*^*(*b-d*)+*y_1_^*^*(*c-d*)+*d*} ≥ {*x_1_y_1_^*^*(*a-b-c+d*)+*x_1_*(*b-d*)+*y_1_^*^*(*c-d*)+*d*}

⇒ *x_1_^*^*[*y_1_^*^*(*a-b-c+d*)-(*d-b*)] ≥ *x_1_*[*y_1_^*^*(*a-b-c+d*)-(*d-b*)]…………..(Eq. A.1)

This inequality has three cases:

(i) *y_1_^*^*(*a-b-c+d*) > (*d-b*) ⇒ *x_1_^*^* = 1 ⇒ *x_2_^*^* = 0………………...(Eq. A.2)

(ii) *y_1_^*^*(*a-b-c+d*) < (*d-b*) ⇒ *x_1_^*^* = 0 ⇒ *x_2_^*^* = 1………………...(Eq. A.3)

(iii) *y_1_^*^*(*a-b-c+d*) = (*d-b*) ⇒ *y_1_^*^* = (*d-b*)/(*a-b-c+d*) & *y_2_^*^* = (*a-c*)/(*a-b-c+d*)...(Eq. A.4)

II. From equations (Eq. 2) and (Eq. 4) (see main text, Results section),

-{*x_1_^*^y_1_^*^*[*k_1_*(*a-b*)+*k_2_*(*d-c*)]+*x_1_^*^*(*k_1_b-k_2_d*)+*y_1_^*^k_2_*(*c-d*)+*k_2_d*}

≥ - {*x_1_^*^y_1_*[*k_1_*(*a-b*)+*k_2_*(*d-c*)]+*x_1_^*^*(*k_1_b-k_2_d*)+*y_1_k_2_*(*c-d*)+*k_2_d*}

⇒ *y_1_^*^*{*x_1_^*^*[*k_1_*(*a-b*)+*k_2_*(*d-c*)]-*k_2_*(*d-c*)} ≤ *y_1_*{*x_1_^*^*[*k_1_*(*a-b*)+*k_2_*(*d-c*)]-*k_2_*(*d-c*)}……(Eq. A.5)

This inequality has three cases:

(i) *x_1_^*^*[*k_1_*(*a-b*)+*k_2_*(*d-c*)] > *k_2_*(*d-c*) ⇒ *y_1_^*^* = 0 ⇒ *y_2_^*^* = 1……………..(Eq. A.6)

(ii) *x_1_^*^*[*k_1_*(*a-b*)+*k_2_*(*d-c*)] < *k_2_*(*d-c*) ⇒ *y_1_^*^* = 1 ⇒ *y_2_^*^* = 0……………..(Eq. A.7)

(iii) *x_1_^*^*[*k_1_*(*a-b*)+*k_2_*(*d-c*)] = *k_2_*(*d-c*) ⇒ *x_1_^*^* = [*k_2_*(*d-c*)]/[*k_1_*(*a-b*)+*k_2_*(*d-c*)] and

*x_2_^*^* = [*k_1_*(*a-b*)]/[*k_1_*(*a-b*)+*k_2_*(*d-c*)]…….(Eq. A.8)

III. Consider (Eq. A.2)

when *x_1_^*^* = 1 and  *x_2_^*^* = 0

Substituting *x_1_^*^* = 1 and  *x_2_^*^* = 0 in (Eq. A.5) we get

*y_1_^*^k_1_*(*a-b*) ≤ *y_1_k_1_*(*a-b*)

This inequality has three cases:

(i) *a = b*  (ii) *a > b* (iii) *a < b*

‘*a*’ is the pay off of bacteria when bacteria thrives only in extracellular compartment and the host attacks bacteria using only extracellular defense mechanisms. ‘*b*’ is the pay off of bacteria when bacteria thrives only in extracellular environment, but the host attacks bacteria using only intracellular defense mechanisms. For obvious reasons, *a = b* and *a > b* are not biologically plausible. However, *b > a* is very likely to happen biologically.

When *b > a*, *y1^*^* = 1 ⇒ *y_2_^*^* = 0

Therefore,

*X^*^*= (*x_1_^*^, x_2_^*^*) = (1, 0) and *Y^*^*= (*y_1_^*^, y_2_^*^*) = (1, 0) is a potential Nash equilibrium

IV. Consider (Eq. A.3)

when *x_1_^*^* = 0 and *x_2_^*^* = 1

Substituting *x_1_^*^* = 0 and *x_2_^*^* = 1 in (Eq. A.5) we get

*y_1_^*^k_2_*(*c-d*) ≤ *y_1_k_2_*(*c-d*)

This inequality has three cases:

(i) *c = d* (ii) *c < d* (iii) *c > d*

‘*c*’ is the pay off of bacteria when bacteria thrives only in intracellular compartment and the host attacks bacteria using only extracellular defense mechanisms. ‘*d*’ is the pay off of bacteria when bacteria thrives only in intracellular environment, but the host attacks bacteria using only intracellular defense mechanisms. For obvious reasons *c = d* and *c* *< d* are not biologically plausible. However, *c > d* is very likely to happen biologically.

When *c > d,*  *y_1_^*^* = 0 ⇒ *y_2_^*^* = 1

Therefore,

*X^*^*= (*x_1_^*^, x_2_^*^*) = (0, 1) and *Y^*^*= (*y_1_^*^, y_2_^*^*) = (0, 1) is a Nash equilibrium

Altogether, there are three potential Nash equilibria in this game:

1. *X^*^*= (*x_1_^*^, x_2_^*^*) = (1, 0) and *Y^*^*= (*y_1_^*^, y_2_^*^*) = (1, 0)

2. *X^*^*= (*x_1_^*^, x_2_^*^*) = (0, 1) and *Y^*^*= (*y_1_^*^, y_2_^*^*) = (0, 1)

3. *X^*^*= (*x_1_^*^, x_2_^*^*) = {[*k_2_*(*d-c*)]/[*k_1_*(*a-b*)+*k_2_*(*d-c*)], [*k_1_*(*a-b*)]/[*k_1_*(*a-b*)+*k_2_*(*d-c*)]}

*Y^*^*= (*y_1_^*^, y_2_^*^*) = {(*d-b*)/[(*a-b*)+(*d-c*)], (*a-c*)/[(*a-b*)+(*d-c*)]}

[From (Eq. A.4) and (Eq. A.8)]
